# Hippuric acid and 3‐(3‐hydroxyphenyl) propionic acid inhibit murine osteoclastogenesis through RANKL‐RANK independent pathway

**DOI:** 10.1002/jcp.28998

**Published:** 2019-07-04

**Authors:** Haijun Zhao, Oxana P. Lazarenko, Jin‐Ran Chen

**Affiliations:** ^1^ Department of Pediatrics University of Arkansas for Medical Sciences Little Rock Arkansas; ^2^ Arkansas Children's Nutrition Center Little Rock Arkansas

**Keywords:** bone resorption, osteoclast, phenolic acid, RANKL

## Abstract

Nutritional factors influence bone development. Previous studies demonstrated that bone mass significantly increased with suppressed bone resorption in early life of rats fed with AIN‐93G semi‐purified diets supplemented with 10% whole blueberry (BB) powder for 2 weeks. However, the effects of increased phenolic acids in animal serum due to this diet on bone and bone resorption were unclear. This in vitro and in ex vivo study examined the effects of phenolic hippuric acid (HA) and 3‐(3‐hydroxyphenyl) propionic acid (3‐3‐PPA) on osteoclastic cell differentiation and bone resorption. We cultured murine osteoclast (macrophage) cell line, RAW 264.7 cells, and hematopoietic osteoclast progenitor cells (isolated from 4‐week‐old C57BL6/J mice) with 50 ng/ml of receptor activator of nuclear factor κ‐Β ligand (RANKL). Morphologic studies showed decreased osteoclast number with treatment of 2.5% mouse serum from BB diet–fed animals compared with those treated with serum from standard casein diet–fed mice in both RAW 264.7 cell and primary cell cultures. HA and 3‐3‐PPA, but not 3–4‐PPA, had dose‐dependent suppressive effects on osteoclastogenesis and osteoclast resorptive activity in Corning osteo‐assay plates. Signaling pathway analysis showed that after pretreatment with HA or 3‐3‐PPA, RANKL‐stimulated increase of osteoclastogenic markers, such as nuclear factor of activated T‐cells, cytoplasmic 1 and matrix metallopeptidase 9 gene/protein expression were blunted. Inhibitory effects of HA and 3‐3‐PPA on osteoclastogenesis utilized RANKL/RANK independent mediators. The study revealed that HA and 3‐3‐PPA significantly inhibited osteoclastogenesis and bone osteoclastic resorptive activity.

## Graphical abstract

We provide evidence for the first time that phenolic hippuric acid (HA) and 3‐(3‐hydroxyphenyl) propionic acid (3‐3‐PPA) had dose‐dependent suppressive effects on osteoclastogenesis and osteoclast resorptive activity in Corning osteo‐assay plates. Inhibitory effects of HA and 3‐3‐PPA on osteoclastogenesis utilized the RANKL/RANK independent mediators.

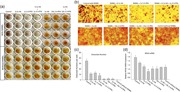

**Abbreviations**
3‐3‐PPA: 3‐(3‐hydroxyphenyl) propionic acid
BB: Blueberry
cAMP: Cyclic adenosine monophosphate
GPR 109A: G‐protein coupled receptor 109 A
HA: Hippuric acid
MMP‐9: Matrix metallopeptidase 9
NF‐κB: Nuclear factor kappa‐light‐chain‐enhancer of activated B cells
NFATc1: Nuclear factor of activated T‐cells, cytoplasmic 1
OPG: Osteoprotegerin
PA: Phenolic acid
RANKL: Receptor activator for nuclear factor kappa‐B ligand
TRAPase: Tartrate‐resistant acid phosphatase

## 1 INTRODUCTION

Physiologic bone development, growth, and repair are coordinated by well‐balanced bone formation and resorption, and this coordination is influenced by factors including nutrition, hormones, and weight bearing/physical activity (Office of the Surgeon General, US, 2004; Shams‐White et al., 2018; Tan et al., 2014). For nutritional factors, consideration has been closely directed to micronutrients, such as calcium, vitamin D, and phosphate, and to macronutrients, such as proteins and fats. Moreover, functional dietary factors from dairy products, fruits and vegetables, and foods contributing to acid‐base balance interact with local bone transcription factors and circulating endogenous hormones to facilitate bone development (Yan et al., 2016). It is of interest to identify specific bioactive compounds. Previous studies have shown that bone mass and size in early pubertal children are significantly dependent on intake of fruits and vegetables (Hardcastle, Aucott, Reid, & Macdonald, 2011; Lanham, 2006; Tylavsky et al., 2004). We have previously described the robust effect of a 10% blueberry (BB)‐supplemented diet on the promotion of bone formation in young male and female rats (Chen et al., 2010). We hypothesized that the significant effects of BB diet on bone mass increase in rapidly growing rats may be associated with increased phenolic acid levels in blood to inhibit osteoclastic bone resorption and to stimulate osteoblast differentiation.

Phenolic acids (PAs), such as hippuric acid (HA) and 3‐(3‐hydroxyphenyl) propionic acid (3‐3‐PPA), are metabolites derived from BB pigment polyphenols that appear in the serum of BB‐fed rats. These molecules have been recently characterized as bioactive in stimulating osteoblast activity and dose‐dependently increasing bone mass in mice (Chen et al., 2014). HA and 3‐3‐PPA are produced by gut microflora through the breakdown of chlorogenic acid and are thereafter absorbed and oxidized in the liver before entering circulation (Marín, Miguélez, Villar & Lombó, 2015). Until recently, these small molecules were not known for functions on stimulating or inhibiting particular cell differentiation or activity; moreover, it has not been proposed that HA and 3‐3‐PPA suppress osteoclastic bone resorption.

Periods of rapid osteoblastic bone formation are essential early in life as well as in adulthood to maintain skeletal health whereas osteoclastic bone resorption is essential to shape and keep an appropriate amount of bone (Clarke, 2008). While osteoclastic bone resorption is an important physiological cellular function in skeletal development, suppression of osteoclastogenesis is effective as a therapeutic approach to bone‐destructive diseases such as osteoporosis and rheumatoid arthritis. Osteoblasts and osteoclasts originate from different lineage of cells. Osteoblasts are derived from mesenchymal stem cells; osteoclasts are derived from hematopoietic lineage (Grigoriadis et al., 2010). Osteoclast differentiation and bone resorptive activities of mature osteoclasts are regulated by series of cellular molecules, such as receptor activator of nuclear factor κ‐Β ligand (RANKL) and macrophage colony‐stimulating factor (Khosla, 2001). RANKL, a secreted protein, is and thought to be produced chiefly by osteoblasts and also by other cell types such as mesenchymal stromal cells, osteocytes, preadipocytes, and chondrocytes (O’Brien, 2010; Wang et al., 2014). Secreted RANKL stimulates receptor activator of nuclear factor kappa‐B (RANK) signaling (Khosla, 2001).

The osteoclastogenic signaling starts from binding of RANKL to its receptor RANK on the surface of osteoclast precursors; this binding triggers the recruitment of tumor necrosis factor (TNF) receptor‐associated factors (Park, Lee, & Lee, 2017). The RANK‐TNF receptor‐associated factor complex activates downstream signal pathways, including the nuclear factor kappa‐light‐chain‐enhancer of activated B cells (NFκB) and mitogen‐activated protein kinase pathways, which lead to the induction and activation of transcription factors such as Fos proto‐oncogene (cFos) and nuclear factor of activated T‐cells, cytoplasmic 1 (NFATc1; Boyce, 2013), resulting in enhanced expression of osteoclast‐specific genes (Kim et al., 2018; Moreaux et al., 2011). In the current report, we hypothesize that natural plant‐derived PAs, HA, and 3‐3‐PPA, directly and transcriptionally inhibit cFos and NFATc1 to decrease osteoclastogenesis via a RANKL‐RANK independent mechanism.

## 2 MATERIALS AND METHODS

### 2.1 Cell cultures

Murine osteoclast progenitor macrophage cell line RAW 264.7 cells were commercially obtained (American Type Culture Collection [ATCC], Manassas, VA, http://www.atcc.org; ATCC^®^ TIB‐71^™^). Nonadherent bone marrow cells from 4‐week‐old female C57BL/J mice were isolated. Bone marrow cells were flushed from femurs and then cultured in T175 flasks (Corning® Cell Culture Flasks; Sigma‐Aldrich) for 2 days to let stromal cells attach. Two days later, nonadherent cells were collected and cultured in appropriate plates with appropriate cell densities. These cells are considered as hematopoietic osteoclast progenitor cells (Chen et al., 2005). Conditional serum for treatment of cells was taken from female rats either fed 10% BB supplemental diet or standard rodent casein diet for 4 weeks as described in our previous study (Chen et al., 2010). Cell cultures were performed in α‐Minimum Essential Medium (Invitrogen, Carlsbad, CA) supplemented with 10% fetal bovine serum (FBS; Hyclone, Logan, UT), penicillin (100 units/ml), streptomycin (100 µg/ml), and glutamine (4 mM). Cells were seeded in 96‐, 12‐, or 6‐well cell culture plates at appropriate density of cells per well for morphology, RNA, and protein expression experiments. At 85% confluence, cells in 96‐well plates were treated with 2.5% rat serum (7.5% FBS) in the presence of 50 ng/ml of soluble RANKL for osteoclastic cell morphologic study.

### 2.2 Osteoclast diﬀerentiation assay, morphologic tartrate‐resistant acid phosphatase staining

RAW264.7 cells or nonadherent bone marrow cells were cultured in 96‐well plates (2 × 10^4^cells/well) in the presence or absence of 50 ng/ml of RANKL. Cells were treated with HA at four different concentrations, 0.01×, 0.1×, 1×, and 10×, with 1× equivalent to concentration of 60 µg/dl of HA free form appeared in blood after 10% BB supplemental diet in rats (Chen et al., 2010). Four different concentrations of 3‐3‐PPA or 3‐(4‐hydroxyphenyl) propionic acid (3–4‐PPA), 0.1×, 1×, 10×, and 100×, with 1× equivalent to concentration of 10 µg/dl of 3‐3‐PPA or 3–4‐PPA free form appeared in blood after 10% BB supplemental diet in rats (Chen et al., 2010). After 4 days for RAW264.7 cell cultures and 5 days for bone marrow cell cultures, the cells were ﬁxed with 4% paraformaldehyde and stained for tartrate‐resistant acid phosphatase (TRAPase) activity using a TRAPase Staining Kit according to the manufacturer's protocols (Sigma‐Aldrich, Acid Phosphatase Leukocyte, Procedure No. 386). TRAP‐positive cells containing >3 nuclei in each well were counted as osteoclasts under an epifluorescent microscope (model BH‐2, Olympus, Imaging America Inc., Center Valley, PA).

### 2.3 Osteoclast resorption activity and proliferation assay

For osteoclast resorption activity assay, RAW264.7 cells or nonadherent bone marrow cells were seeded in six‐well collagen‐coated plates (BD Biosciences) at a density of 1 × 10^5^ cells/well, and cells were treated with 50 ng/ml RANKL for 2–3 days. When osteoclasts begin to differentiate to mature cells on day 3, the cells were dissociated and the same number of osteoclastic cells were cultured onto hydroxyapatite‐coated plates (CLS3989; Corning). The cells were treated with different concentrations of HA, 3‐3‐PPA, and 3–4‐PPA, or 2.5% conditional serum for another 48 hr in the presence or absence of RANKL. Cells in the culture plates were fixed using 2.5% glutaraldehyde with or without Von Kossa staining. The areas of hydroxyapatite resorption were observed by light microscopy and analyzed using the Image J software (imagej.nih.gov/ij/). Cell viability was measured by 3‐(4,5‐dimethylthiazol‐2‐yl)−2,5‐diphenyltetrazolium bromide (MTT) assay. Briefly, fixed cells in the culture plates were washed with phosphate‐buffered saline and incubated with MTT (0.5 mg/ml) at 37°C for 3 hr. After rinsing out MTT, 100 µl of dimethylformamide was used to dissolve the reduced formazan crystals, and microplate reader was used to determine the absorbance of each well at 540 nm.

### 2.4 RNA isolation, real‐time reverse transcription‐polymerase chain reaction

RAW264.7 cells were cultured in 12‐well plates (1.2 × 10^5^ cells/well) with or without RANKL (50 ng/ml) in the presence or absence of 1× HA for 1, 2, 3, or 4 days. RNA from cultured cells were extracted using TRI Reagent (MRC Inc., Cincinnati, OH) according to the manufacturer's recommendation followed by DNase digestion and column cleanup using QIAGEN mini columns (Chen et al., 2016). Reverse transcription was carried out using an iScript cDNA Synthesis Kit from Bio‐Rad (Hercules, CA). Real‐time reverse transcription‐polymerase chain reaction (RT‐PCR) was carried out using SYBR Green and an ABI 7000 Sequence Detection System (Applied Biosystems, Foster City, CA); gene expression data were normalized by housekeeping gene glyceraldehyde 3‐phosphate dehydrogenase (Chen et al., 2016). All primers for RT‐PCR analysis used were designed using Primer Express Software 2.0.0 (Applied Biosystems) and are listed in Table 1.

**Table 1:** Real‐time reverse transcription‐polymerase chain reaction (RT‐PCR) primer sequences

Gene	Forward primer	Reverse primer
NFκB	CCTCTGGCGAATGGCTTTAC	GCTATGGATACTGCGGTCTGG
NFATc1	GATCCCGTTGCTTCCAGAAAAT	TCTGTCTCCCCTTTCCTCAGCT
MMP9	TCTTCTGGCGTGTGAGTTTCCA	TGCACTGCACGGTTGAAGCAAA
Cathepsin K	GTGGGTGTTCAAGTTTCTGC	GGTGAGTCTTCTTCCATAGC
GPR109A	CGCTGCCTTCGAAAGAAAAC	GCCCCTGGAATACTTCTGGTT
RANK	TCGTCCACAGACAAATGCAAAC	TGGAAGAGCTGCAGACCACAT
β‐Catenin	GATATTGACGGGCAGTATGCAA	AACTGCGTGGATGGGATCTG
Cmyc	GCTCTCCATCCTATGTTGCGG	TCCAAGTAACTCGGTCATCATCT
Traf6	AAAGCGAGAGATTCTTTCCCTG	ACTGGGGACAATTCACTAGAGC
GAPDH	GTATGACTCCACTCACGGCAAA	GGTCTCGCTCCTGGAAGATG

Abbreviations: GAPDH, glyceraldehyde 3‐phosphate dehydrogenase; MMP9, matrix metallopeptidase 9; NF‐κB, nuclear factor kappa‐light‐chain‐enhancer of activated B cells; NFATc1, nuclear factor of activated T‐cells, cytoplasmic 1; RANK, receptor activator of nuclear factor kappa‐B.

### 2.5 Western blotting

RAW264.7 cells were cultured in six‐well plates (3.5 × 10^5^cells/well) with or without RANKL (50 ng/ml) in the presence or absence of 1× HA or 3‐3‐PPA for 3 days. Total protein extracts were prepared using radioimmunoprecipitation assay buffer (Solarbio). Western blots were performed using standard protocols (Chen, Lazarenko, Blackburn, & Shankar, 2017). The protein lysates were quantiﬁed and separated by sodium dodecyl sulfatepolyacrylamide gel electrophoresis. Lysates were then transferred to polyvinylidene ﬂuoride membranes (Millipore). Immunoblotting with primary antibodies (NFATc1 [SAB2101576; Sigma‐Aldrich, St. Louis, MO], cFos [ab134122; Abcam, Cambridge, MA], matrix metallopeptidase 9 [MMP9; sc‐6840; Santa Cruz, Dallas, TX], Cathepsin K [sc‐48353; Santa Cruz, Dallas, TX], GPR109A [sc‐134583; Santa Cruz, Dallas, TX], β‐catenin [#610154; BD Transduction Lab], pGSK3β [#9336; Cell Signaling], α Tubulin [sc‐12462; Santa Cruz, Dallas, TX], and β‐Actin [A1978; Sigma‐Aldrich, St. Louis, MO]) at 1:1000 dilution and then by the corresponding horseradish peroxidase (HRP) conjugated secondary antibodies followed. Blots were developed using PIERCE Biotechnology Chemiluminescence Kits. Using a ProteinSimple FluorChem E System (San Jose, CA), bands of interest were visualized and imaged under chemiluminescent detection. The intensity of the bands in the autoradiograms was quantitated using a VersaDoc^TM^ Imaging System (Bio‐Rad).

### 2.6 GPR109A transfection, intracellular cyclic adenosine monophosphate level measurement using ELISA

Cell cultures were similar to above described but different treatment times, cultured RAW264.7 cells (six‐well plates, 3.5 × 10^5^ cells/well) were treated with 1× HA for 1, 2, 3 or 4 days with or without RANKL (50 ng/ml). Cells were transfected with either overexpression plasmid GPR109A (#MC211930; HCAR2, Origene), or shRNA GPR109A (#TR511005; HCAR2‐sh, Origene), or control vector (PCMV6KN, Origene) using Lipofectamine 3000. 1× overexpression plasmid transfection equal to DNA concentration 0.4 pmol/µl, and 1× short hairpin RNA (shRNA) equal to 12.5 ng/µl of RNA as suggested by the manufactory. Cell culture medium and cell lysates were collected for measurements of cyclic adenosine monophosphate (cAMP) levels in cell culture medium or intracellular. Direct immunoassay cAMP assay (ab65355; Abcam) was performed following the manufacturer's recommendations. The assay anchors cAMP polyclonal antibodies onto recombinant Protein G‐coated 96‐well plates. cAMP‐HRP conjugate directly competes with cAMP from binding to the cAMP antibody on the plate. After incubation and washing the plates, HRP activity was determined using a plate reader set at OD450 nm.

### 2.7 Statistical analyses

All analyses used Stata 12.0 (Stata Corporation, College Station, TX) and Prism 5 (GraphPad, San Diego, CA). Data are expressed as the mean ± standard error; n equals to the number of samples/group. Differences within groups were evaluated using *t* test or one‐way analysis of variance followed by Tukey's post hoc test with *p* < .05 considered signiﬁcant. Representative images from three separate cell culture experiments are displayed. Significant dose‐ or time‐dependent effects of tested compounds were assessed using Cruick's nonparametric test for trend (Cuzick, 1985).

## 3 RESULTS

### 3.1 HA and 3‐3‐PPA dose‐dependently inhibit osteoclastogenesis in RAW264.7 cells and nonadherent mouse bone marrow cells

**Figure 1:**
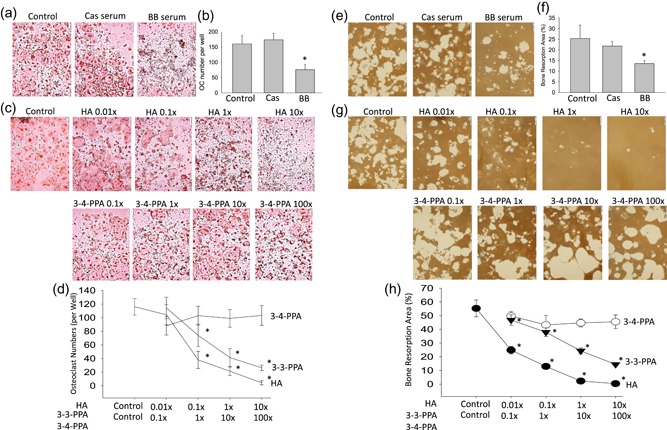
HA and 3‐3‐PPA dose‐dependently inhibit osteoclastogenesis: in vitro RAW 264.7 cells. (a) RAW 264.7 cells were treated with 2.5% serum either from standard casein diet–fed (Cas serum) or blueberry diet–fed (BB serum) mice in the presence of 50 ng/ml RANKL for 5 days. Pictures showing osteoclast morphology after TRAPase staining, control, 2.5% FBS. (b) Osteoclast number per well with triplicates for each treatment. (c) RAW 264.7 cells were treated with four different concentrations of HA (hippuric acid), or 3–4‐PPA, or 3‐3‐PPA (data not show because they similar to HA) in the presence of 50 ng/ml RANKL for 5 days. Pictures showing osteoclast morphology after TRAPase staining. (1× HA = 60 µg/dL, 1× 3‐3‐PPA or 3–4‐PPA = 10 µg/dL). (d) Osteoclast number per well with triplicates for each treatments. (e) RAW 264.7 cells were cultured for osteoclast resorptive activity in Corning osteo‐assay plates. Pictures showing resorption pits on Von Kossa staining after treatments. (f) Percentage of bone resorption area per well with triplicates for each treatments. (g) Pictures showing resorption pits on Von Kossa staining after cells treated with HA or 3–4‐PPA. (H) Percentages of bone resorption pits per well with triplicates for each treatments. Independent experiments were repeated more than three times, obtaining similar results each time, *p < .05, significant differences versus control by *t* test, dose response was assessed using Cruzick's nonparametric test for trend. FBS, fetal bovine serum; RANKL, receptor activator of nuclear factor κ‐Β ligand; TRAPase, tartrate‐resistant acid phosphatase [Color figure can be viewed at wileyonlinelibrary.com]

HA and 3‐3‐PPA are PAs. BB diet is associated with high concentrations of these PAs in animal blood; their structures have been characterized previously (Chen et al., 2010). RAW 264.7 cells were treated with 2.5% serum either from standard casein diet–fed mice (Cas serum) or BB diet–fed mice (BB serum) in the presence of 50 ng/ml RANKL for 5 days. Osteoclast morphology after TRAPase staining (Figure 1a), and osteoclast numbers (Figure 1b) were significantly decreased in 2.5% BB serum–treated wells. Although previous studies demonstrated high concentrations of PAs, HA, and 3‐3‐PPA appeared in serum from 10% BB‐fed animals (Chen et al., 2010), and they are bioactive to stimulate osteoblast activity, it was unclear how those increased PAs have effects on bone, particularly on osteoclastogenesis and bone resorption. RAW 264.7 cells were treated with four different concentrations of HA, 3‐3‐PPA, or 3–4‐PPA in the presence of 50 ng/ml RANKL for 5 days. Photomicrographs in Figure 1c display representative osteoclast morphology after TRAPase staining (3‐3‐PPA data not shown because it was similar to HA) (1×HA = 60 µg/dl, 1×3‐3‐PPA, or 3–4‐PPA = 10 µg/dl). Osteoclast number per well, with triplicates for each treatment, showed dose‐dependent decreases in HA‐ and 3‐3‐PPA‐treated cells, but not in 3–4‐PPA‐treated cells (Figure 1d). RAW 264.7 cells were cultured for evaluation of osteoclast resorptive activity in Corning osteo‐assay plates. Figure 1e presents representative resorption pits in white on Von Kossa staining after treatments with control FBS only, serum from standard diet animals (Cas serum), or serum from BB diet animals (BB serum). BB serum significantly inhibited bone resorption area (Figure 1f, percentage of bone resorption area per well with triplicates for each treatment). Figure 1g shows representative osteoclast resorption pits in white on Von Kossa staining after treatments with four different concentrations of HA, 3‐3‐PPA, or 3–4‐PPA in the presence of 50 ng/ml RANKL (3‐3‐PPA data not shown because there were similar to HA). Percentage of bone resorption pits per well with triplicates for each treatment showed dose‐dependent decreases in osteoclastic bone resorption in HA‐ and 3‐3‐PPA‐treated wells, but not in 3–4‐PPA‐treated wells (Figure 1h).

**Figure 2:**
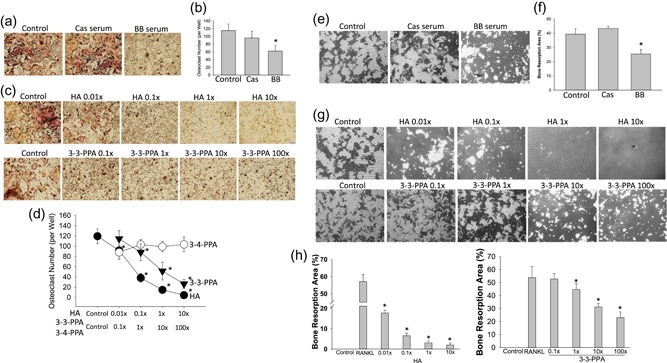
Effects of HA and 3‐3‐PPA on inhibiting osteoclastogenesis in nonadherent bone marrow cells from 4‐week‐old wild type C57Bl mice. (a) Bone marrow cells were isolated from 3 4‐week‐old wild type mice, and suspended for 48 hr, nonadherent bone marrow cells were re‐cultured and treated with 2.5% serum either from standard casein diet–fed (Cas serum) or blueberry diet–fed (BB serum) mice in the presence of 50 ng/ml RANKL for 5 days. Pictures showing osteoclast morphology after TRAPase staining, control, 2.5% FBS. (b) Osteoclast number per well with triplicates for each treatment. (c) Nonadherent bone marrow cells from wild type mice were treated with four different concentrations of HA (hippuric acid), or 3–4‐PPA (data not show because no effects), or 3‐3‐PPA in the presence of 50 ng/ml RANKL for 5 days. Pictures showing osteoclast morphology after TRAPase staining. (1× HA = 60 µg/dL, 1× 3‐3‐PPA or 3–4‐PPA = 10 µg/dL). (d) Osteoclast numbers per well with triplicates from cultures of nonadherent bone marrow cells from wild type mice with indicated treatments. (e) Nonadherent bone marrow cells from wild type mice were cultured for osteoclast resorptive activity in Corning osteo‐assay plates. Pictures showing resorption pits after treatments without Von Kossa staining. (f) Percentage of bone resorption area per well with triplicates for each treatments. (g) Pictures showing resorption pits after cells wild type treated with HA or 3‐3‐PPA in the presence of RANKL (without Von Kossa staining). (h) Percentages of bone resorption pits per well of cells from wild type with triplicates for each treatments. Independent experiments were repeated more than three times, obtaining similar results each time, *significant differences versus control by *t* test, dose response were assessed using Cruzick's nonparametric test for trend. RANKL, receptor activator of nuclear factor κ‐Β ligand [Color figure can be viewed at wileyonlinelibrary.com]

Bone marrow cells were isolated from 4‐week‐old three wild type mice and suspended for 48 hr; nonadherent bone marrow cells were re‐cultured and treated with FBS, 2.5% Cas serum or BB serum, plus four different concentrations of HA, or 3‐3‐PPA, or 3–4‐PPA in the presence of 50 ng/ml RANKL for 5 days. We recapitulated the RAW264.7 cell results in ex vivo nonadherent bone marrow cell cultures. BB serum significantly inhibited osteoclastogenesis in hematopoietic nonadherent bone marrow cells (Figure 2a,b) and osteoclast resorptive activity in Corning osteo‐assay plates (Figure 2e,f). HA and 3‐3‐PPA, but not 3–4‐PPA dose‐dependently inhibited osteoclastogenesis (Figure 2c,d) and osteoclastic bone resorption in Corning osteo‐assay plates (Figure 2g,h).

### 3.2 Inhibition of osteoclastogenic signaling by HA and 3‐3‐PPA in RAW264.7 cells

**Figure 3:**
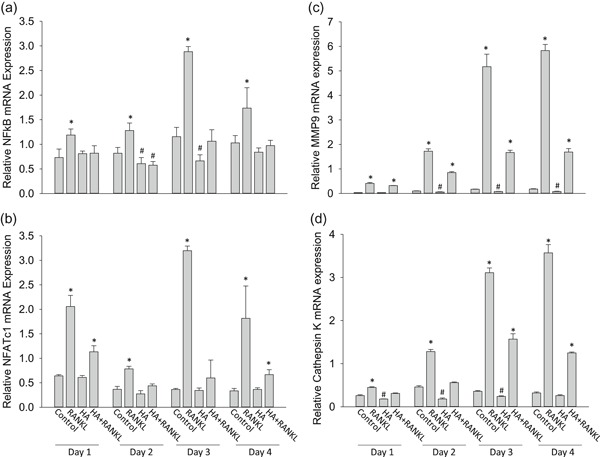
HA suppresses osteoclastogenic gene expression in RAW264.7 cells. Real‐time PCR shows: (a) relative NF𝑘BmRNA expression; (b) relative NFATc1 mRNA expression; (c) relative MMP9 mRNA expression; (d) relative Cathepsin K mRNA expression in RAW 264.7 cells treated with RANKL (50 ng/ml), HA (1×) and the combination of HA and RANKL, control = PBS treated for 4 indicated days. *^,#^Significant differences versus control by *t* test. Data are means ± standard error with triplicates. HA, hippuric acid; MMP9, matrix metallopeptidase 9; mRNA, messenger RNA; NF‐κB, nuclear factor kappa‐light‐chain‐enhancer of activated B cells; NFATc1, nuclear factor of activated T‐cells, cytoplasmic 1; RANKL, receptor activator of nuclear factor κ‐Β ligand

To study molecular signaling pathways that are involved in actions of HA and 3‐3‐PPA on inhibiting osteoclastogenesis, we cultured RAW264.7 cells and treated cells with or without 1× HA in the presence or absence of RANKL for 1, 2, 3, and 4 days. Real‐time PCR shows relative NFκB and NFATc1 gene expression were significantly higher in cells treated with RANKL (50 ng/ml) at each time point, as expected (Figure 3a,b). HA completely blocked RANKL‐induced NFκB expression at each time point (Figure 3a). On days 2 and 3, HA inhibited NFκB expression significantly down to basal level (untreated control; Figure 3a). HA completely blocked RANKL‐induced NFATc1 gene expression on days 2 and 3 (Figure 3b). MMP9 and Cathepsin K are considered as two downstream osteoclastogenic markers, and as expected, RANKL time‐dependently increased MMP9 and Cathepsin K expression (Figure 3c,d). Interestingly, time‐dependent, RANKL‐induced MMP9 and Cathepsin K gene expression were significantly inhibited by HA (Figure 3c,d). Moreover, on days 2, 3, and 4, compared to untreated control, HA significantly inhibited MMP9 gene expression (Figure 3c), and HA significantly inhibited Cathepsin K gene expression below to basal level (untreated control) on days 1, 2, and 3 (Figure 3d).

**Figure 4:**
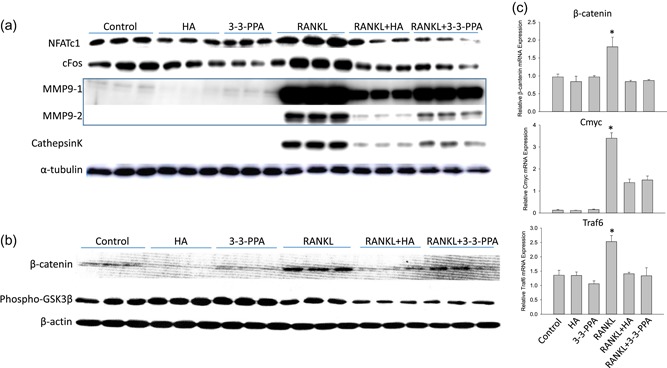
HA and 3‐3‐PPA inhibit RANKL‐induced osteoclastogenic signaling. (a) Western blot shows RANKL activated NFATc1, cFos, MMP9 and Cathepsin K protein expression after RAW264.7 cells were treated for 3 days. Both 1× HA and 10× 3‐3‐PPA inhibited those RANKL‐induced protein expression. MMP9‐1 and MMP9‐2 are same membrane but different exposure time. α‐tubulin is a protein loading control. (b) Western blot analysis shows β‐catenin and GSK3beta expression in RAW264.7 cells after 4 days treatment with either 1× HA, 10× 3‐3‐PPA, RANKL or their combinations, β‐actin is a protein loading control. (c) real‐time PCR shows mRNA expression of β‐catenin and downstream genes Cmyc and Traf6 in RAW264.7 cells after 4 days treatment with either 1× HA, 10× 3‐3‐PPA, RANKL or their combinations, GAPDH was used as a housekeeping gene. *p < .05 versus control by *t* test. GAPDH, glyceraldehyde 3‐phosphate dehydrogenase; HA, hippuric acid; MMP9, matrix metallopeptidase 9; mRNA, messenger RNA; RANKL, receptor activator of nuclear factor κ‐Β ligand

RAW264.7 cells were cultured in six‐well plates and cells were treated with or without 1× HA or 1× 3‐3‐PPA in the presence or absence of RANKL for 3 days. Proteins were isolated for Western blot analysis. It is very clear that RANKL increased NFATc1, cFos, MMP9, and Cathepsin K protein expression (Figure 4a). Compared to untreated control, HA and 3‐3‐PPA inhibited NFATc1, cFos, MMP9, and Cathepsin K protein expression (Figure 4a). It is even more clear that both HA and 3‐3‐PPA significantly inhibited RANKL‐induced NFATc1, cFos, MMP9, and Cathepsin K protein expression (Figure 4a). There are not many signaling pathways that could regulate both osteoblast and osteoclast activity, however, β‐catenin is one of these signaling molecules. We therefore next checked if HA and 3‐3‐PPA regulate β‐catenin signaling in pre‐osteoclasts. We found both HA and 3‐3‐PPA blocked RANKL‐induced β‐catenin protein overexpression (Figure 4b), phosphorylation of GSK3β were lower in either HA or 3‐3‐PPA‐treated with RANKL in RAW264.7 cells (Figure 4b). Consistent with Western blot data, real‐time PCR showed that HA and 3‐3‐PPA blocked RANKL‐induced β‐catenin and its downstream target gene expression such as Cmyc and Traf6 (Figure 4c).

**Figure 5:**
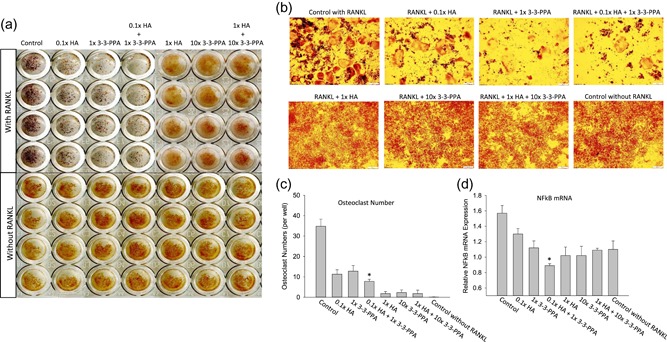
Synergetic effects of HA and 3‐3‐PPA on osteoclastogenesis. (a) TRAPase staining of original culture plates of RAW264.7 cells treated with two different doses of either HA or 3‐3‐PPA, or with their combinations in presence or absence of RANKL in four wells per treatment. (b) pictures showing osteoclast morphology after TRAPase staining from a typical area of a well from each treatments (top row from a). (c) osteoclast numbers per well with quadruplicates from cultures of RAW264.7 cells with indicated treatments. (d) real‐time PCR shows NFκB gene expression from cultures of RAW264.7 cells with indicated treatments in quadruplicates. *p < .05 versus either 0.1× HA or 1× 3‐3‐PPA treatment by *t* test. HA, hippuric acid; NF‐κB, nuclear factor kappa‐light‐chain‐enhancer of activated B cells; RANKL, receptor activator of nuclear factor κ‐Β ligand; TRAPase, tartrate‐resistant acid phosphatase [Color figure can be viewed at wileyonlinelibrary.com]

HA and 3‐3‐PPA were found two predominant PAs in blood in animals fed a BB diet, it was suggested by our previous study that they have synergetic effect on promoting osteoblastic cell activity (Chen et al., 2014), whether they have additive effects on inhibiting osteoclast activity are not clear. RAW264.7 cells were treated with HA plus 3‐3‐PPA in two different individual doses in the presence of RANKL. Due to significant activities of HA and 3‐3‐PPA at doses of 1× and 10× on inhibiting osteoclast formation ( Figure 5a), HA and 3‐3‐PPA in this combination did not show additive effect on inhibiting osteoclast formation (Figure 5a,b,c). However, when cells were treated with 0.1× HA plus 1× 3‐3‐PPA, osteoclast morphology (osteoclast formation) (Figure 5a,b,c) and osteoclastic cell differentiation gene (Figure 5d, NFkB showed as example) expression were significantly inhibited compared to each individual treatments, indicating HA and 3‐3‐PPA have synergetic effects on inhibiting osteoclast activities.

### 3.3 HA and 3‐3‐PPA inhibit GPR109A expression to increased intracellular cAMP in RAW264.7 cells

**Figure 6:**
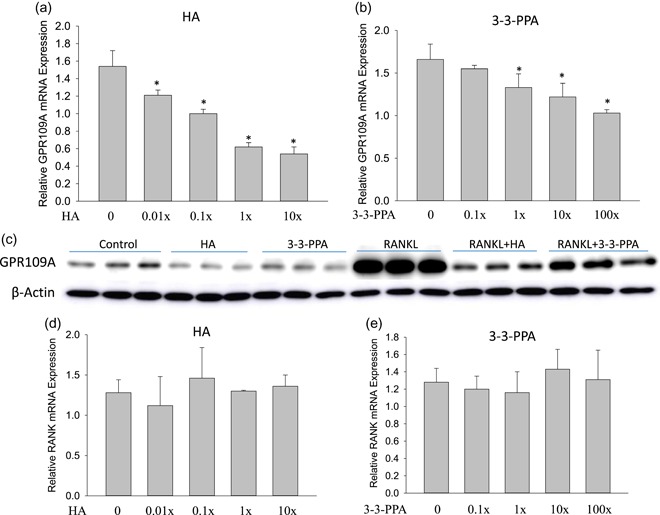
HA and 3‐3‐PPA inhibit GPR109A expression in RAW264.7 cells. Real‐time PCR shows both HA (a) and 3‐3‐PPA (b) dose‐dependently suppressed GPR109A gene expression in RAW264.7 cells. *p < .05 versus control by *t* test, dose response were assessed using Cruzick's nonparametric test for trend. (c) Western blot analysis shows RANKL activated GPR109A protein expression after RAW264.7 cells were treated for three days. Both 1× HA and 3‐3‐PPA inhibited RANKL‐induced GPR109A protein expression. β‐actin is a protein loading control. Real‐time PCR shows both HA (d) and 3‐3‐PPA (e) do not change RANK gene expression in RAW264.7 cells. HA, hippuric acid; RANK, receptor activator of nuclear factor kappa‐B; RANKL, receptor activator of nuclear factor κ‐Β ligand [Color figure can be viewed at wileyonlinelibrary.com]

**Figure 7:**
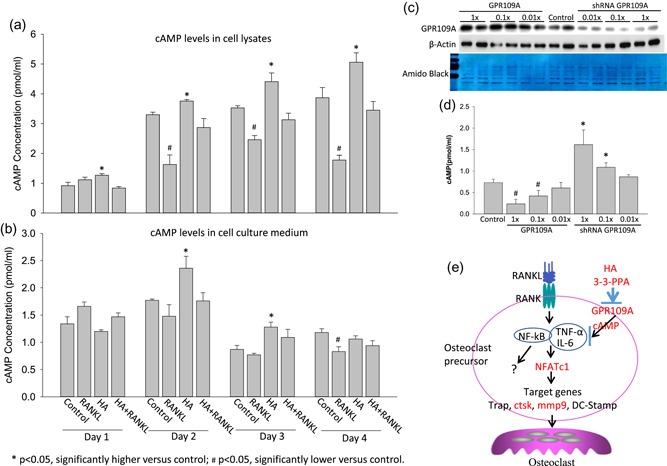
HA and 3‐3‐PPA change intracellular cAMP levels in RAW264.7 cells. RAW 264.7 cells were treated with RANKL (50 ng/ml), HA (1×) and the combination of HA and RANKL, control = PBS for 4 indicated days. (a) Intracellular cAMP levels from cell lysates were measured using an ELISA method. (b) cAMP levels in cell culture medium were measured using an ELISA method. (c) Western blots showing GPR109A expression in RAW 264.7 cells were transfected with GPR109A overexpression plasmid (three different concentrations, 1× equal to 0.4 pmol/µl of DNA as suggested by manufactory), or shRNA GPR109A (three different concentrations, 1× equal to 12.5 ng/µl of RNA as suggested by manufactory), or control vector. β‐actin and Amido Black showing lording control. (d) Intracellular cAMP levels after GPR109A overexpression plasmid or shRNA GPR109A. (e) Working hypothesis of HA or 3‐3‐PPA inhibits GPR109A expression leads to cAMP accumulation in the cytoplasm to interfere osteoclastogenic signaling. *^,#^Significant differences versus control by *t* test. Data are means ± standard error with triplicates, time‐dependent changes were assessed using Cruzick's nonparametric test for trend. cAMP, cyclic adenosine monophosphate; HA, hippuric acid; IL, interleukin; RANKL, receptor activator of nuclear factor κ‐Β ligand; shRNA, short hairpin RNA; TNF, tumor necrosis factor [Color figure can be viewed at wileyonlinelibrary.com]

HA and 3‐3‐PPA are known structurally similar to nicotinic acid or niacin (vitamin B3), and nicotinic acid is reported to bind to GPR109A (Goel & Dunbar, 2016). GPR109A is abundantly expressed in macrophages and in mature osteoclast (Feingold, Moser, Shigenaga, & Grunfeld, 2014; GPR109A BioGPS). Therefore, we targeted on G‐protein coupled receptor (GPR) 109A expression to search for possible membrane receptor‐mediated actions of HA and 3‐3‐PPA on osteoclastogenesis inhibition. RAW264.7 cells were treated with four different concentrations of HA or 3‐3‐PPA. Surprisingly, HA and 3‐3‐PPA both dose‐dependently suppressed GPR109A gene expression (Figure 6a,b). GPR109A protein expression levels were also investigated, and we found both HA and 3‐3‐PPA suppressed GPR109A protein expression (Figure 6c). Notably, RANKL robustly increased GPR109A expression (Figure 6c). Consistent with inhibitory effects of HA and 3‐3‐PPA on osteoclastogenic markers, HA and 3‐3‐PPA significantly inhibited RANKL‐induced GPR109A protein expression in RAW264.7 cells (Figure 6c). Unfortunately, we did not found any differences in RANK gene expression between any concentrations of HA or 3‐3‐PPA treatment of RAW264.7 cells and untreated control (Figure 6d,e). GPR109A is a Gi‐G protein coupled receptor, and it has been shown that GPR109A reduces accumulation or inactivates intracellular cAMP (Gaidarov et al., 2013; Li et al., 2011). We finally examined if HA and 3‐3‐PPA change intracellular second messenger cAMP levels in RAW364.7 cells. RAW264.7 cells were treated with or without 1× HA in the presence or absence of RANKL for 1, 2, 3, and 4 days. Intracellular and cell culture medium cAMP levels were measured by a recently developed and now commercially available ELISA method. RANKL alone significantly decreased intracellular levels of cAMP on days 2, 3, and 4 (Figure 7a). Intracellular cAMP showed a time‐dependent increase in cells treated with HA alone (Figure 7a). With the combination of HA and RANKL treatments, intracellular cAMP levels were back to control levels at each time point (Figure 7a). cAMP levels in cell culture medium displayed a different pattern than they are intracellular: we found increased cAMP levels in HA‐treated cells compared to those untreated controls on Day 2 and 3 (Figure 7b). cAMP levels in culture medium were significantly decreased in RANKL‐treated wells on Day 4 (Figure 7b). When RAW364.7 cells were transfected with GPR109A overexpression plasmid (Figure 7c) (three different concentrations, 1× equal to 0.4 pmol/µl of DNA as suggested by manufactory), intracellular cAMP levels were found significantly lower (Figure 7d). On the other hand, when RAW364.7 cells were transfected with shRNA GPR109A (Figure 7c; three different concentrations, 1× equal to 12.5 ng/µl of RNA as suggested by manufactory), intracellular cAMP levels were found significantly higher (Figure 7d). These data indicate that HA or 3‐3‐PPA inhibits GPR109A expression leads to cAMP accumulation in the cytoplasm to interfere osteoclastogenic signaling (Figure 7e).

## 4 DISCUSSION

Our novel data suggest direct effects of PAs, HA, and 3‐3‐PPA on inhibiting osteoclastogenesis through a RANKL‐RANK independent mechanism. It is known that RANKL is a type II transmembrane protein produced mainly by osteoblasts and other cell types including mesenchymal stromal cells, osteocytes, preadipocytes, and chondrocytes (O’Brien, 2010; Wang et al., 2014). It is cleaved by proteases to yield soluble form RANKL. Secreted RANKL in turn stimulates pre‐osteoclastic RANK signaling (Feng & Teitelbaum, 2013) and is able to directly induce the differentiation of precursor cells, such as bone marrow‐derived macrophages, into mature and active osteoclasts (Feng & Teitelbaum, 2013). RANKL has its specific decoy receptor, osteoprotegerin (OPG), on the osteoclastic cell surface. After interacting with OPG, RANKL binds to RANK and hence to increase osteoclastogenesis and bone resorption. Using both osteoclastic cell line RAW 264.7 cells and primary nonadherent bone marrow cells, we have shown that HA and 3‐3‐PPA dose‐dependently inhibited osteoclast differentiation and osteoclast resorptive activity. Both HA and 3‐3‐PPA did not effect on OPG or RANK expression; therefore, their inhibitory effects on osteoclastogenesis do not utilize RANKL/OPG/RANK mediators. Our data suggest that HA or 3‐3‐PPA inhibits pre‐osteoclast surface GPR109A expression, thus increasing intracellular second messenger cAMP level to suppress expression of osteoclast‐specific genes and osteoclast differentiation. The inhibitory effects of HA and 3‐3‐PPA on this osteoclastogenic pathway has never been proposed before. We did not observe effect of 3–4‐PPA within dosages tested on osteoclastogenesis. The structural difference between 3‐3‐PPA and 3–4‐PPA is hydroxyl group in different position of phenyl, we don’t know exactly how to explain why 3‐3‐PPA, but not 3–4‐PPA, inhibits osteoclast formation, but possibly is due to different activities binding to GPR109A.

Throughout bone development and remodeling periods, osteoblasts and osteoclasts have opposing tasks. During physiologic bone development to build optimal bone mass in early life, osteoblastic bone formation is more favorable than osteoclastic bone resorption. During this period, osteoclasts as a bone‐specific macrophage primarily monitor appropriate bone shaping by controlling both bone resorption and formation. Yet, the required optimal and relative numbers of osteoclasts and their activities for this process are unknown. Enhanced bone resorption by osteoclasts not compensated by osteoblast bone formation may result in pathological bone diseases (e.g., osteoporosis, rheumatoid arthritis, Paget's disease, periodontal disease, and multiple myeloma). Identifying novel molecules that can regulate osteoclastogenesis or inhibit osteoclast activity has been an important clinical goal. It is also important to identify food‐based approaches to improve bone health and optimize bone growth. We have previously reported that BB diet suppressed bone resorption by downregulating RANKL expression in stromal cells and/or peroxisome proliferator‐activated receptor γ in preadipocytes. We suggested that bone marrow stromal cells are targets for BB diet to regulate RANKL expression and osteoclast differentiation in young rapidly growing rats. In addition, we have also recently reported on the effect of HA on osteoblastic bone formation, and together to our current findings, it appears that HA and 3‐3‐PPA have effects on anti‐osteoclastic cell resorption. Clearly, more in vivo studies are needed, in additional models such as ovariectomized mouse models or collagen‐induced rheumatoid arthritis animal models to comprehensively test the hypothesis that HA and 3‐3‐PPA have value to inhibit bone resorption.

We have previously described that BB diet contains abundant bioactive compounds derived from polyphenols, which mainly include proanthocyanidins, anthocyanidins, flavones, phenolic acid, and stibenes. PAs are efficiently absorbed and oxidized in the liver and are one of the final metabolites derived from the polyphenols in vivo. HA and 3‐3‐PPA were identified two phenolic acid metabolites derived from BB pigment polyphenols that appear in the serum of BB diet–fed rats with the highest concentrations (Chen et al., 2010). Using HPLC/MS, we found that their concentrations were increased with increased percentage of BB consumption in the diet (Zhang et al., 2013). HA and 3‐3‐PPA inhibited RANKL‐induced osteoclast differentiation in a significant dose‐dependent manner. The inhibitory eﬀects of HA and 3‐3‐PPA were not due to any obvious toxic eﬀects on RAW264.7 cells. Although their synergistic effect will be tested in future studies, HA and 3‐3‐PPA each signiﬁcantly suppressed the expression of osteoclastogenesis‐related marker genes and proteins. HA and 3‐3‐PPA significantly inhibited NFATc1 expression, with a subsequent reduction in expression of downstream osteoclastogenic marker genes. As a required protein for RANKL‐induced osteoclast formation, a highly phosphorylated form of NFATc1 is in the cytoplasm (Sheridan, Heist, Beals, Crabtree, & Gardner, 2002), and its expression is regulated by cFos (Mohamed et al., 2007). Following interactions between RANKL and RANK, NFATc1will be activated and enters into the nucleus to stimulate osteoclast‐specific gene expression, including integrin β3, Acp5 (TRAcP), and Ctsk (Crotti et al., 2008, Matsumoto et al., 2004). It has been reported that severe bone sclerosis observed in cFos mutant mice is due to the blockage of osteoclast formation (Matsuo et al., 2000). In addition, it is well recognized that RANKL‐RANK interaction results in TNF receptor‐associated factor 6 (TRAF6) recruitment and nuclear factor kappa‐B, c‐Jun amino‐terminal kinase, extracellular signal‐regulated kinase, and p38 signaling pathways activation (Jules, Ashley, & Feng, 2010). However, further investigations are required to determine if HA and 3‐3‐PPA inhibit osteoclast differentiation through these sub‐signaling pathways.

To provide evidence of HA and 3‐3‐PPA inhibiting osteoclastogenesis through mediators different than RANK in cell membrane of pre‐osteoclasts, we examined GPR109A expression during osteoclast differentiation. We previously determined that HA and 3‐3‐PPA bind to GPR109A, a G‐protein coupled receptor (Ren et al., 2009; Tunaru et al., 2003). Herein, we were surprised to find that both HA and 3‐3‐PPA significantly inhibited GPR109A gene expression in pre‐osteoclasts in the absence or presence of RANKL. Evidence suggested that activation of GPR109A results in reduced cAMP levels which may affect activity of cAMP‐dependent protein kinase A and phosphorylation of target proteins, leading to neutrophil apoptosis (Insel, Zhang, Murray, Yokouchi, & Zambon, 2012). We found that GPR109A gene expression is inhibited by HA and 3‐3‐PPA resulting in increased levels of intracellular cAMP. It is unknown if the decreased expression of osteoclastic gene markers is the result of a cell type‐specific phenomenon or increased levels of intracellular cAMP in pre‐osteoclasts by HA and 3‐3‐PPA. Intracellular cAMP is known as an important second messenger that mediates a diverse set of extracellular signals, and GPRs evoke cAMP‐mediated signaling via Gs (a guanine nucleotide‐binding protein related to adenylyl cyclase activation). It was reported that stimulation of cAMP/PKA signaling suppressed osteoclast differentiation (Weivoda et al., 2016).

In summary, we demonstrated for the first time that HA and 3‐3‐PPA, PAs found in the highest concentrations in blood from mice fed a BB diet, dose‐dependently inhibited osteoclastogenesis through a RANKL/RANK independent mechanism to protect against increased osteoclastic bone resorption. Our data suggested that the effect of HA and 3‐3‐PPA on osteoclastogenesis is through modulating cell membrane GPR109A to control cAMP levels in cytoplasm and hence osteoclastogenic gene expression. Our current research provides first‐ever insights into mechanisms driving the effects of food‐derived PAs HA and 3‐3‐PPA to regulate osteoclastogenesis and bone resorption.

## AUTHOR CONTRIBUTIONS

H.Z. contributed in the performance of experiments; O.P.L. performed the cell, biochemical, and molecular experiments; J‐R.C. designed and performed the study and wrote the paper. All authors edited the manuscript.
